# Pediatric respiratory infectious emergencies: clinical profiles and outcomes

**DOI:** 10.25122/jml-2024-0044

**Published:** 2024-07

**Authors:** Imad Mohammed Khojah, Noura Wagih Alazhary, Anas Saeed Alyazidi, Mohammed Abdullah Alsubaie, Maha Khider Alghamdi, Abdulkarim Abbas Jawhari

**Affiliations:** 1 Department of Emergency Medicine, King Abdulaziz University, Jeddah, Saudi Arabia; 2 Department of Pediatrics, Dr. Soliman Fakeeh Hospital, Jeddah, Saudi Arabia; 3 Faculty of Medicine, King Abdulaziz University, Jeddah, Saudi Arabia; 4 Department of Internal Medicine, King Faisal Specialist Hospital and Research Center, Jeddah, Saudi Arabia; 5 Department of Pediatrics, King Abdulaziz University Hospital, Jeddah, Saudi Arabia

**Keywords:** Pediatric infectious diseases, respiratory tract infections, emergency department, pediatric, children, Saudi Arabia

## Abstract

Infectious diseases are among the most frequent causes of hospital admission and a substantial contributor to morbidity and mortality. These diseases pose a persistent concern, particularly within the pediatric population, where delays or inappropriate management can result in serious harm. Additionally, infectious diseases contribute to overcrowding in pediatric emergency departments (EDs). This study aimed to explore the epidemiology, clinical presentation, diagnostics, outcome, and social and behavioral impacts of infectious diseases on child health. We conducted a retrospective, single-hospital study at a tertiary care center that is publicly funded and owned to serve the entire community. Pediatric patients with at least one or more chief complaints related to the respiratory system or infectious etiology were included. Data analysis was performed using SPSS to assess relationships between variables. A total of 15,106 patients were included, with a mean age of 3.80 years. The largest age group was toddlers (42.8%). Most cases (71.9%) were classified as urgent (Priority 3). Regarding patient outcomes, 76.1% were discharged after receiving appropriate treatment in the ED, and 22.9% required admission for further management. Nearly 38% of patients presented with a single complaint. Fever was the most frequent complaint across all groups. In summary, this study provides insights into the presentation of pediatric respiratory infectious diseases in the emergency room. The study revealed that toddlers were the most affected age group, with fever and cough being the common symptoms.

## INTRODUCTION

Infectious diseases are among the most frequent causes of hospital admission and significantly contribute to morbidity and mortality [1,2]. Recently, the world has been witnessing a wave of severe infectious disease outbreaks, often with devastating consequences [1]. There is an enduring concern about these diseases and their impact on the pediatric population, where delays or inappropriate management may lead to serious harm [3]. Furthermore, the global burden of infectious diseases contributing to child mortality remains considerable and is complicated by increasing antimicrobial resistance and the discovery of newer strains and viruses [4]. Recent studies estimate an annual burden of 10 million deaths among children associated with infectious diseases [4]. It was also found that infectious disease can contribute to 12.5% of infant deaths [5], 10% of deaths among toddlers, and 5% of those aged 5–14 years [6]. Moreover, studies have concluded that these diseases contribute to overcrowding in pediatric emergency departments (ED) [7].

Despite the importance of clinical assessment in such cases and the need to identify signs and symptoms that can help clinicians differentiate between common presenting complaints, there is a lack of sufficient data in this area. Studies have found that when data on pediatric infectious diseases are provided, particularly concerning their etiology, clinical presentation, and outcomes, substantial advances can be made in this field due to the preventable nature of these conditions [8]. Therefore, in this study, we aimed to explore the epidemiology, clinical presentation, diagnostics, outcomes, and social and behavioral impacts of infectious diseases on child health.

## MATERIAL AND METHODS

### Study design and setting

**Figure 1:**
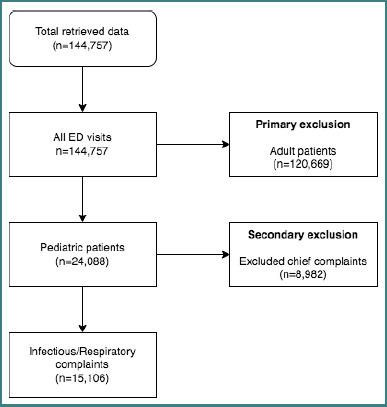
Flowchart of sampling methodology

Following the Strengthening the Reporting of Observational Studies in Epidemiology (STROBE) reporting guideline for cross-sectional studies [9], we conducted this retrospective, single-hospital study. The study was conducted at King Abdulaziz University Hospital, a tertiary care center that is publicly funded and owned to serve the entire community with a bed capacity of 750 beds and up to 900 beds in an emergency setting, receiving an estimated 60,000 visits annually. It has specialized units, services, and isolation wards for pediatric diseases. The data were directly extracted from the administrative electronic health records of the emergency department. All emergency department visits were extracted from January 1–December 31, 2022. A total of 144,757 visits, including adults and pediatrics, were filtered to include pediatric patients (<14 years old), yielding 24,088 visits. The remaining visits were filtered according to their presenting chief complaints, which resulted in the inclusion of a total of 15,106 visits (Figure 1). Data were also grouped according to the age group of patients as follows: A) neonates (birth–1 month), B) infants (1 month–1 year), C) toddler (1-3 years), D) preschool (3-6 years), E) school age (6–12 years), (F) adolescent (12–18 years) using the standardized labeling according to the clinical practice of the center. The demographic data of each patient, including their gender, nationality, triage priority, provisional diagnosis, clinical outcome, chief complaint, associated complaints, length of stay (LOS), laboratory investigations (i.e., X-ray, CT scan, MRI, lab workup), and the number of medications, were retrieved. The symptoms and conditions were defined by a validated list of International Classification of Diseases (ICD-10) codes. We implemented the Australasian Triage Scale (ATS) methodology to assign triage levels, aligning with our center’s standardized protocols and guidelines. These protocols categorize patients based on the urgency of their medical needs, ranging from 1 (requiring immediate resuscitation) to 4 (less urgent conditions) and 5 (non-urgent cases), ensuring both patient safety and optimal allocation of resources. Our study included pediatric patients presenting with one or more chief complaints related to the respiratory system or infectious conditions. Additionally, we included cases where patients received a provisional diagnosis related to respiratory issues, regardless of whether their complaint was constitutional or contributed to their respiratory condition. The following were the criteria for exclusion: adult patients >14 years old, patients with non-infectious and non-respiratory-related chief complaints, and patients with incomplete clinical or demographic data.

### Statistical analysis

Statistical analysis was conducted using IBM SPSS Statistics version 27 (IBM Corp., Armonk, NY, USA) to assess relationships between variables. The normality of data distribution was evaluated using the Shapiro-Wilk test and histograms. Variables were categorized as qualitative and quantitative. Qualitative variables were summarized with frequencies and percentages (%), while quantitative data were expressed as mean ± standard deviation (SD). Statistical significance was set at *P* < 0.05, and results were reported with a 95% confidence interval (CI)..

## RESULTS

### Demographic characteristics and clinical data

A total of 15,106 patients were included in our study, with a mean age of 3.80 years and a standard deviation of 3.72. Male subjects comprised 55.1% of the study population. The largest age group was toddlers, comprising 42.8% of the participants. Most patients were Saudi nationals (72.6%). Regarding the triage level, most cases (71.9%) were classified as urgent (Priority 3). X-rays were the most frequently ordered imaging modality, with 5,505 X-rays performed. Regarding patient outcomes, 76.1% were discharged after receiving appropriate treatment in the ED, and 22.9% required admission for further management.

When comparing the years under study, 2019 had the highest number of ED visits, with 6,424 recorded. In 2020, the patients visiting the ED were relatively older, with an average age of 4.10 years and a standard deviation of 3.96. Additionally, 2020 had the largest number of cases classified as resuscitation (Priority 1) based on their triage level. Over the years, there has been a decrease in non-urgent cases, with 19, 6, and 2 cases reported in 2019, 2020, and 2021, respectively. Table 1 shows the detailed patients’ demographics and clinical data according to the year of their visit.

**Table 1:** Demographic characteristics and clinical data of pediatric patients visiting the emergency department in 2019-2021

	2019 (*n* = 6424)	2020 (*n* = 3614)	2021 (*n* = 5068)	Total (*n* = 15,106)
Age (years), Mean ± SD	3.78 ± 3.64	4.10 ± 3.96	3.62 ± 3.63	3.80 ± 3.72
**Age groups**				
Infant (< 1 year)	1077 (16.8)	585 (16.2)	855 (16.9)	2517 (16.6)
Toddler (1-3 years)	2718 (42.3)	1452 (40.2)	2294 (45.3)	6464 (42.8)
Pre-school (4-6 years)	1227 (19.1)	651 (18)	931 (18.4)	2809 (18.6)
School-age (7-12 years)	1236 (19.2)	765 (21.2)	829 (16.4)	2830 (18.7)
Adolescent (13-18 years)	166 (2.6)	161 (4.5)	159 (3.1)	486 (3.2)
**Sex**				
Male	3517 (54.8)	2060 (57)	2734 (54)	8318 (55.1)
Female	2907 (45.2)	1554 (43)	2334 (46)	6788 (44.9)
**Most frequent nationalities**				
Saudi Arabian	4794 (74.6)	2414 (66.8)	3755 (74.1)	10963 (72.6)
Yemeni	441 (6.9)	322 (8.9)	326 (6.4)	1089 (7.2)
Indian	214 (3.3)	121 (3.3)	86 (1.7)	421 (2.8)
Pakistani	150 (2.3)	111 (3.1)	95 (1.9)	356 (2.4)
Egyptian	149 (2.3)	103 (2.9)	69 (1.4)	321 (2.1)
**Triage level**				
Priority 1 - Resuscitation	72 (1.1)	79 (2.2)	57 (1.1)	208 (1.3)
Priority 2 - Emergent	1230 (19.1)	791 (21.9)	1115 (22)	3136 (20.8)
Priority 3 - Urgent	4594 (71.5)	2499 (69.1)	3772 (74.4)	10865 (71.9)
Priority 4 - Less Urgent	509 (7.9)	239 (6.6)	122 (2.4)	870 (5.8)
Priority 5 - Non-Urgent	19 (0.3)	6 (0.2)	2 (0.1)	27 (0.2)
**Imaging ordered**				
X-ray	1791 (27.9)	1696 (46.9)	2018 (39.8)	5505 (36.4)
CT scan	201 (3.1)	189 (5.2)	197 (3.9)	587 (3.9)
MRI	10 (0.2)	16 (0.4)	11 (0.2)	37 (0.2)
**Outcome**				
Discharged	5316 (82.7)	2492 (69)	3693 (72.9)	11501 (76.1)
Admitted	1057 (16.5)	1077 (29.8)	1331 (26.3)	3465 (22.9)
Discharged against medical advice	28 (0.4)	34 (0.9)	37 (0.7)	99 (0.7)
Transferred to another hospital	21 (0.3)	6 (0.2)	2 (0.1)	29 (0.2)
Deceased	4 (0.1)	4 (0.1)	4 (0.1)	12 (0.1)

Data are presented as frequency and (%), and age is presented as mean and standard deviation

### Number of emergency department visits

From 2019 to 2021, there was a fluctuation in the annual number of visits to the pediatric ED for infectious-related complaints, with a decrease from 6,424 in 2019 to 3,614 in 2020, followed by an increase to 5,068 in 2021.

**Figure 2:**
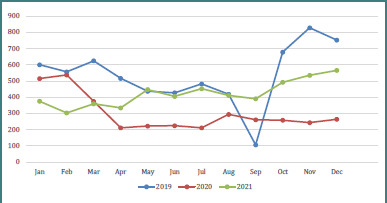
The number of pediatric emergency department visits related to infectious complaints per month between 2019 and 2021

The number of pediatric ED visits related to infectious complaints per month from 2019 to 2021 is presented in Figure 2. During 2019, there was a decreasing trend in the monthly number of visits, reaching a low point of only 106 visits in September. Subsequently, there was a marked increase, with the highest monthly visit count of 828 visits recorded in November. In 2020, the monthly number of visits decreased from February to April, after which it plateaued for the remainder of the year, with an average of 243 visits per month. In contrast to 2019, the monthly number of visits in 2021 showed an increasing trend, reaching a peak of 565 visits at the end of the year.

### Patients’ complaints

**Figure 3:**
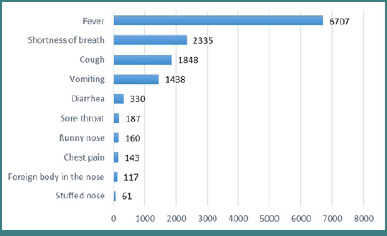
The most common infectious-related complaints among pediatric patients who visited the emergency department between 2019 and 2021

Figure 3 displays the most frequent infectious-related complaints reported by pediatric patients who visited the ED. Of these, 38% presented with a single complaint, while 39.5% and 18% presented with two and three complaints. Among these complaints, fever was the most frequent, accounting for 44.4% of cases, followed by shortness of breath (15.5%), cough (12.2%), and vomiting (9.5%).

When categorized by age groups (Table 2), fever was the most frequent complaint across all groups, while the second and third most common complaints varied by age group. Infants and toddlers had shortness of breath and cough as the second and third most common complaints, whereas preschool-age children reported cough and vomiting. School-age children and adolescents reported shortness of breath and vomiting as the second and third most common complaints. Along with the chief complaints, children also presented with associated complaints such as abdominal pain (7.5%), edema (1.5%), rash (1.1%), and fatigue (0.9%), as presented in Table 3.

**Table 2:** The most frequent infectious-related complaints of pediatric patients visiting the emergency department in 2019-2021, categorized by age groups

Complaints, *n* (%)	Infant (< 1 year)	Toddler (1-3 years)	Pre-school (4-6 years)	School-age (7-12 years)	Adolescent (13-18 years)	Total
Fever	878 (36.9)	3231 (54.5)	1284 (53.8)	1135 (50.7)	179 (45.7)	6707 (50.3)
Shortness of breath	757 (31.8)	928 (15.6)	274 (11.5)	301 (13.4)	75 (19.1)	2335 (17.5)
Cough	370 (15.5)	837 (14.1)	328 (13.8)	277 (12.4)	36 (9.2)	1848 (13.9)
Vomiting	215 (9.1)	548 (9.3)	327 (13.7)	301 (13.4)	47 (12.0)	1438 (10.8)
Diarrhea	73 (3.1)	182 (3.1)	36 (1.5)	32 (1.4)	7 (1.8)	330 (2.5)
Sore throat	6 (0.3)	31 (0.5)	61 (2.6)	74 (3.3)	15 (3.8)	187 (1.4)
Runny nose	44 (1.9)	72 (1.2)	21 (0.9)	21 (1.0)	2 (0.5)	160 (1.2)
Chest pain	3 (0.1)	8 (0.2)	18 (0.7)	86 (3.8)	28 (7.1)	143 (1.1)
Foreign body in the nose	0 (0)	79 (1.3)	30 (1.3)	6 (0.3)	2 (0.5)	117 (0.9)
Stuffed nose	30 (1.3)	17 (0.2)	7 (0.2)	6 (0.3)	1 (0.3)	61 (0.4)

* Adolescents aged 15-18 years were excluded.

**Table 3:** The most frequent associated complaints of pediatric patients presenting with infectious-related complaints in the emergency department in 2019-2021

Associated Complaints	*n* (%)
Abdominal pain	1137 (7.5)
Edema	228 (1.5)
Rash	162 (1.1)
Fatigue	143 (0.9)
Headache	123 (0.8)
Earache	91 (0.6)
Dizziness	77 (0.5)
Constipation	72 (0.5)
Jaundice	67 (0.4)
Pallor	59 (0.4)
Abdominal distension	58 (0.4)

## DISCUSSION

The present study sheds light on the clinical profiles and outcomes of pediatric infectious emergencies, aiming to provide valuable insights and a better understanding of their triage level. Our analysis focused on pediatric patients presenting with infectious emergencies, highlighting their implications for clinical practice and guiding future research efforts. Throughout the study, fever was the predominant symptom among pediatric patients (44.4%). This finding aligns closely with the patterns observed in prior investigations, which consistently identify fever as the most common complaint among children and infants arriving at emergency departments [10,11]. This prevalence can be attributed to several factors. Children have developing immune systems that are more susceptible to infections than adults. Their frequent interactions in school or daycare settings and close contact with peers and various surfaces increase their exposure to pathogens, heightening their susceptibility to infections and subsequent fevers.

We observed a substantial decrease in pediatric infectious emergency visits in 2020, as shown in Figure 2. This is consistent with previous studies [12,13] reporting a decrease in pediatric emergency department visits from 2019 to 2020. That might be attributed to the impact of the COVID-19 pandemic. Emergency room visits may have decreased significantly as a result of the patients’ and their parents’ fear of visiting due to lockdown measures, potential virus exposure, and public health advisories. School closures and reduced social interactions further limited children’s exposure to common infectious agents. Moreover, increased parental focus on managing minor illnesses at home likely contributed to decreased pediatric emergency department visits [14].

Effective management of pediatric patients seeking care in the ED depends on pediatric emergency department triage. Different triage systems have been developed and assessed for reliability and validity and are used by emergency departments around the world to evaluate the severity of incoming patients’ conditions and assign treatment priorities, such as the Emergency Severity Index (ESI), the Manchester Triage System (MTS), the Canadian Triage and Acuity Scale (CTAS), and the Australasian Triage Scale (ATS) [15]. The Australasian Triage Scale (ATS), used in our study, is a widely used pediatric triage system validated and proven reliable in pediatric emergency departments [16]. Level 1 is the most urgent and requires immediate care and treatment, while level 5 is the least urgent. In emergencies, where every second counts, using a triage system is crucial to ensure patients receive the right care at the right time. Thus, the increase in level 1 cases in our study in 2020 could be due to the predisposing factors mentioned above regarding COVID-19 precautions; therefore, patients may present late to the emergency department [17]. Triage systems have an effective system for managing patients, allocating resources, controlling infections, and ultimately improving patient outcomes. Early detection and isolation of potentially contagious individuals are crucial in preventing further transmission of infectious diseases, especially those that might spread quickly. The risk of disease spreading within the ED is decreased with appropriate identification and isolation of infectious cases through triage, as well as identifying high-risk patients such as infants, immunocompromised children, or those with underlying medical conditions that make them more vulnerable to infectious diseases.

It is important to acknowledge the limitations of the study, as it is a descriptive study and cannot establish the direct cause and effect of different factors. The lack of power analysis in the sampling is also a contributor. Also, there can be a possibility of finding some data collection inaccuracies because the descriptive study mainly depends on self-reported measures. These limitations were addressed by including all the reported cases.

## CONCLUSION

This study provides detailed insights into the complaints and diagnoses associated with pediatric infectious diseases in the ED. The findings revealed that toddlers were the most affected age group, with fever and cough being the most prevalent symptoms. The study highlights the significant proportion of patients requiring admission, indicating the severity of the diseases and the delayed presentations. Furthermore, the impact of the COVID-19 pandemic on ED visits was emphasized, with a noticeable decrease in visits during that period. These findings underscore the importance of maintaining access to emergency care during public health emergencies and the need for ongoing monitoring of ED presentation trends. The study contributes to our understanding of the epidemiology of pediatric infectious illnesses, offering valuable guidance for clinical decision-making and public health initiatives. However, to further enhance this research, conducting more comprehensive studies involving multi-center data, diverse study designs, varied geographic areas, and different COVID-19 precautions is recommended. Such studies would enable a more accurate exploration of the associations between different factors, complementing the findings presented in this paper.
